# Influence of Pr Content on Structural Evolution of Doped Ceria-Based High-Entropy Oxides

**DOI:** 10.3390/molecules31040598

**Published:** 2026-02-09

**Authors:** Dalibor Tatar, Jakov Babić, Stjepan Šarić, Jelena Kojčinović, Petra Šušak, Anamarija Stanković, Laura Milišić, Andraž Mavrič, Cora Deák, Gergő Ballai, Imre Szenti, Ákos Kukovecz, Igor Djerdj

**Affiliations:** 1 Department of Chemistry, Josip Juraj Strossmayer University of Osijek, Cara Hadrijana 8/A, HR-31000 Osijek, Croatia; dtatar@kemija.unios.hr (D.T.); jakov.babic@kemija.unios.hr (J.B.); stjepan.saric@kemija.unios.hr (S.Š.); jbijelic@kemija.unios.hr (J.K.); petra.susak@kemija.unios.hr (P.Š.); aster@kemija.unios.hr (A.S.); 2 Materials Research Laboratory, University of Nova Gorica, Vipavska 13, SI-5000 Nova Gorica, Slovenia; laura.milisic@ung.si (L.M.); andraz.mavric@ung.si (A.M.); 3 Department of Applied and Environmental Chemistry, University of Szeged, Rerrich Béla Sq. 1, H-6720 Szeged, Hungary; corapravda@gmail.com (C.D.); ballaigergo@gmail.com (G.B.); szentiimre@gmail.com (I.S.); kakos@chem.u-szeged.hu (Á.K.)

**Keywords:** ceria, high-entropy oxides, methylene blue, praseodymium, structural evolution, wastewater treatment

## Abstract

High-entropy fluorite oxides offer exceptional tunability of structure and functionality through controlled multi-cation substitution. In this work, Ce-Zr-Pr-Sm-Eu-based high-entropy oxides, with systematically varied Pr content, were synthesized using a modified sol–gel citrate method to investigate the influence of Pr incorporation on lattice structure, defect formation, and photocatalytic performance. All compositions crystallized in a single-phase cubic fluorite structure, where increasing Pr concentration induced gradual lattice expansion and microstrain due to the substitution of larger Pr^3+^ ions. Morphological and surface analyses revealed porous nanostructures at moderate Pr levels, while excessive Pr promoted densification and reduced surface accessibility. Spectroscopic studies confirmed the coexistence of Pr^3+^/Pr^4+^ and Ce^3+^/Ce^4+^ redox couples, strong 4*f*–2*p* orbital hybridization, and enhanced defect-related electronic states that narrowed the optical bandgap. The optimized Pr-doped composition exhibited almost 100% degradation of methylene blue under UV light over 30 min, untypical for semiconductors with a narrower bandgap, and is enabled by efficient charge separation and redox cycling between Ce and Pr centers.

## 1. Introduction

Advanced functional materials, defined by their multi-component compositions, represent a rapidly developing field in material science. They are well known as high-entropy materials defined by their multicomponent composition, typically incorporating five or more cations in near-equimolar ratios within a single crystallographic position [1,2,3]. Their stability is primarily regulated by a large configurational entropy, which reduces the Gibbs free energy and suppresses phase segregation at higher temperatures [4]. This concept, initially adapted from high-entropy alloys to oxide systems by Rost et al. [5], in 2015 enabled the formation of single-phase solid solutions from typically incompatible binary oxides through entropy-driven stabilization [6]. The intrinsic structural disorder resulting from random cation distribution in high entropy oxides gives rise to properties unattainable in conventional ordered oxides [7,8]. Higher configuration entropy, combined with lattice distortion and charge compensation, produces complex defect chemistry that directly influences ionic transport, redox activity, and thermal stability [9,10]. These disorder-driven effects grant high-entropy oxides enhanced tolerance to thermal cycling and superior oxygen mobility as compared to pure oxides, making them promising materials for different applications [11,12,13,14]. Among them, fluorite-type oxides, typically derived from CeO_2_, ZrO_2_, or mixed rare-earth oxides exhibits outstanding chemical and thermal stability. Their ability to accommodate large cationic size and valence mismatches without structural collapse enables a disordered but coherent oxygen sublattice, which supports defect mediated oxygen transport with tunable redox behavior [15,16].

Cerium oxide serves as an ideal structural foundation for high-entropy design due to its cubic fluorite type lattice and exceptional redox flexibility [17]. The reversible Ce^4+^/Ce^3+^ redox couple enables oxygen storage and release under changing oxygen partial pressures [18]. When Ce^4+^ is partially reduced to Ce^3+^, charge neutrality is maintained through oxygen vacancy formation, which controls oxygen migration and surface reactivity [19]. Such vacancies improve ionic conductivity, catalytic performance, and oxygen storage capacity, all of which can be further tuned through controlled doping strategies. Aliovalent doping with rare-earth cations, such as Sm^3+^, Gd^3+^, Nd^3+^, Y^3+^, introduces charge compensating oxygen vacancies and lattice distortion, reducing defect formation energy and enhancing ion mobility [20]. The high substitutional tolerance of CeO_2_ and its ability to maintain the crystal structure even under severe redox cycling make it ideal “host” for entropy-stabilized solid solutions [15]. Among various dopants, Pr^4+/3+^ is known as particularly effective dopant, since its substitution generates oxygen vacancies [21]. At moderate dopant concentrations, Pr lower vacancy formation energies and increase surface reducibility, due to mixed valence state enables redox cycling and enhances oxygen storage capacity [22]. However, excessive doping often leads to enhanced cation mobility and sintering, resulting in grain coalescence and reduced surface area [23].

Despite progress in understanding the defect chemistry of rare-earth doped ceria, the relationship between dopant composition, oxygen non-stoichiometry, and surface textural evolution, particularly within high-entropy frameworks, remains insufficiently understood [14,24,25,26]. While most research has focused on the electronic and catalytic properties of rare-earth doped ceria, comprehensive insights into how these dopants influence surface area, porosity, and sintering kinetics are still lacking [27,28,29,30]. Notably, the unique redox flexibility of Pr^4+/3+^ introduces fundamentally distinct pathways for oxygen retention, vacancy clustering, and structural compaction. In response, this study seeks to elucidate the interconnected effects of Pr doping on the structural, surface, and compositional characteristics of CeO_2_-based high-entropy oxides. Analyzed compounds were systematically synthesized and tested, with varying Pr content and thoroughly analyzed to establish quantitative correlations between dopant-induced lattice distortion, oxygen vacancy formation, and surface changes, further identifying optimal dopant levels that maximize active sites while minimizing densification. These findings advance the understanding of the defect–structure–property relationship in high-entropy oxides and their suitability for advanced applications. Furthermore, the synthesized compounds were evaluated as photocatalysts for methylene blue degradation to provide deeper insights into the structure–property–application relationship.

Photocatalysis emerged as a powerful advanced oxidation process for environmental applications, enabling the degradation of persistent organic pollutants through light-driven redox reaction on semiconductor surface [31]. Upon irradiation with photons possessing energy equal or greater than the bandgap of the semiconductor, electron-hole pairs are generated, initiating a cascade of surface reactions that leads to the formation of highly reactive oxygen species, which are capable of mineralizing organic contaminants [32]. Among various organic dyes, methylene blue is widely employed as a model pollutant in photocatalytic research, due to its well defined molecular structure, strong visible-light adsorption, and chemical stability [33]. Recent studies demonstrated that defect engineering, introduction of oxygen vacancies and mixed valence cationic states are factors that can precisely tune the properties of semiconductor material to enhance photocatalytic activity [34,35]. Oxygen vacancies introduce localized electronic states within a bandgap, which facilitates charge trapping and transfer processes, simultaneously alongside promoting surface adsorption and activation of molecular oxygen [35]. In ceria-based photocatalysts, the main advantage is the reversible Ce^3+/4+^ redox pair, which enables efficient oxygen storage and release as well as enhanced ROS generation under irradiation. When combined with additional redox-active dopants, synergistic effects can arise through coupled redox cycling and defect stabilization [36].

## 2. Results and Discussion

### 2.1. Structural Characterization of the Material

#### 2.1.1. Powder X-Ray Diffraction

**Figure 1:**
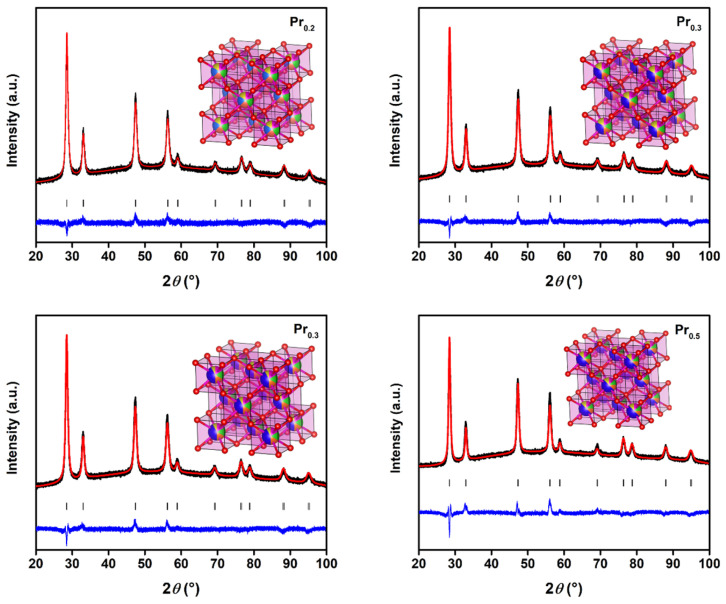
Rietveld output plot of synthesized high-entropy oxides, with visualized structure shown in the insets of each plot. Black line represents experimental plot, red line calculated plot, black points represent Braggs positions, while blue line represents difference plot. In visualized structures, oxygen anions are depicted with red, while cations are depicted in different colors, with Pr depicted in blue.

PXRD combined with Rietveld refinement (Figure 1) confirmed that all Pr-substituted high-entropy oxides crystallize in a single-phase cubic fluorite structure (*Fm*–3*m*), as evidenced by the excellent fitting quality (*R*_wp_ = 10–17%, *GoF* < 1.8) and absence of any secondary impurities. The refinement results are summarized in Table S1.

The lattice parameters increase slightly from *a* = 5.4205 Å for Pr_0.2_ to *a* = 5.4376 Å for Pr_0.5_, accompanied by a monotonic expansion of the unit cell volume from 159.26 to 160.78 Å^3^. This systematic lattice dilation indicates the successful incorporation of Pr ions into the cationic sublattice and correlates with the larger ionic radius of Pr^3+^ (1.126 Å) compared to Ce^4+^ (0.97 Å) and Zr^4+^ (0.84 Å). The trend follows Vegard’s law, suggesting the formation of a homogeneous solid solution without phase segregation. The average microstrain and average apparent crystallite size derived from peak broadening reveal a nanoscale size (10–12 nm) and moderate lattice strain (0.28–0.35%). The strain reflects local distortions induced by multivalent Pr species (Pr^3+/4+^) and the resulting formation of oxygen vacancies. These intrinsic defects and local charge imbalances contribute to static lattice disorder, a structural feature typical for high-entropy fluorite oxides. Such disorder increases with configurational complexity and enhances the defect tolerance of the lattice, stabilizing the cubic fluorite structure even at higher Pr concentrations. The gradual lattice expansion and strain modulation with increasing Pr content suggests competition between the substitutional incorporation of larger Pr^3+^ ions and the formation of charge-compensating oxygen vacancies.

#### 2.1.2. Scanning Electron Microscopy

**Figure 2:**
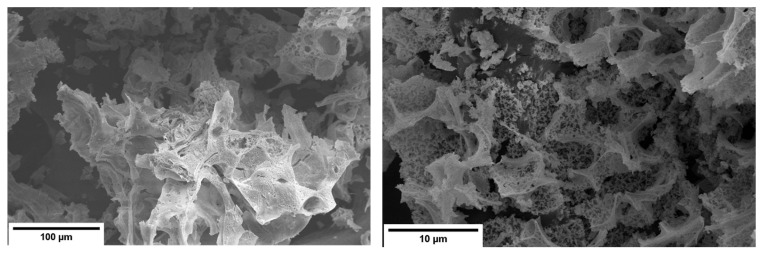
SEM images at different magnifications for a Pr_0.2_ compound.

The SEM images reveal porous, sponge or sheet-like microstructure, consisting of interconnected, irregular agglomerates and open macropores, as seen in Figure 2. Such morphology is characteristic of materials obtained using sol–gel citrate-nitrate methods, where the decomposition of organic precursors and the evolution of gaseous species during heating and calcination generates a lightweight, foam like oxide network. At higher magnifications, the samples display rough, fragmented surfaces composed of thin sheet-like structures and open intergranular cavities. This morphology indicates incomplete densification and limited particle coalescence, even after calcination, which can be attributed to the presence of different mixed-valence species and oxygen-vacancy-induced lattice distortions. These defects reduce cation diffusivity and impede grain boundary mobility, thereby stabilizing a fine and highly porous microstructure [37]. The hierarchical porosity and rough surface topology are expected to increase the accessible surface area and enhance oxygen transport within the oxide matrix [38,39].

#### 2.1.3. Physisorption Analysis

Introduction of trivalent rare earth cations into the CeO_2_ fluorite type lattice typically influences the materials structural, textural and redox characteristics. In our research, Pr possesses larger ionic radius than Ce^4+^, which induces local lattice distortions and generates compensating oxygen vacancies. These structural changes increase defect-related surface activity, directly enhancing adsorption–desorption performance, which is responsible for the catalytic properties of the materials. Experimental results from the present work confirm that all synthesized compounds exhibit type IV nitrogen adsorption–desorption isotherms with H3 hysteresis loops [40], classifying them as mesoporous materials with slit-like pore geometries (Figure S1). Measured specific surface areas of the Pr-doped compounds range between 25.9 m^2^g^−1^ for Pr_0.5_ and 33.8 m^2^g^−1^ for Pr_0.2_, in contrast to 21.5 m^2^g^−1^ for pure CeO_2_, as shown in Table S2. The enhancement at lower Pr molar concentration can be attributed to the formation of additional surface defects (e.g., oxygen vacancies) and disordered domains, thereby increasing the density of surface-active sites. This aligns with prior studies on Pr-doped ceria, which reported that moderate Pr incorporation promotes oxygen mobility and surface redox activity without significant structural collapse [41]. However, a higher dopant concentration usually induces sintering and particle agglomeration during calcination, reducing accessible surface area and pore volume. The enhanced diffusivity of Pr^3+^ ions at higher concentration is known to facilitate early-stage sintering by grain boundary diffusion, consistent with observations from Shajahan et al. [23]. The reduction in pore size at higher dopant concentration, particularly Pr_0.5_ is also consistent with well-known behavior of rare-earth modified systems, where the dopant cations occupy both bulk and near-surface sites, reducing the interconnectivity of the pores. The densification effect suppresses mesopore formation, shifting pore distribution towards smaller diameters and limiting gas diffusion through solid matrix [42].

### 2.2. Spectroscopic Characterization of the Material

#### 2.2.1. Energy Dispersive X-Ray Spectroscopy

**Figure 3:**
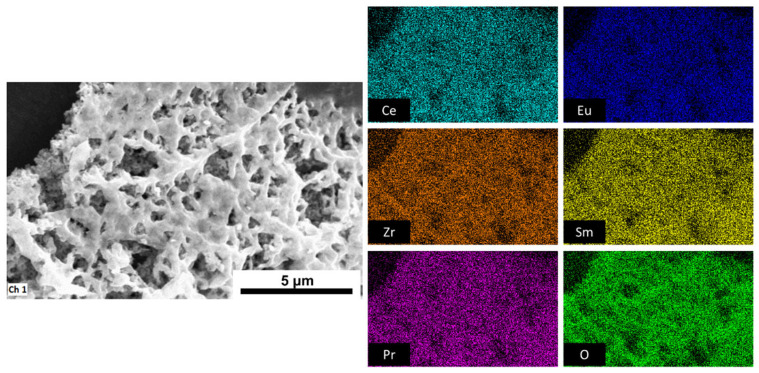
EDS mapping showing uniform distribution of the elements, confirming formation of high-entropy oxides.

EDS confirmed the successful incorporation of all intended cations (Ce, Zr, Sm, Eu, and Pr) into a single-phase structure without any evidence of elemental segregation or phase separation. The elemental mapping demonstrated a homogeneous distribution of all constituents across the analyzed regions (Figure 3), indicating that the compounds exhibit compositional homogeneity characteristics of HEOs. Quantitative EDS results from Table S3 revealed that experimentally determined cation ratios closely matched their theoretical stoichiometries, with only minor deviations, which are due to energy overlap, and additionally confirmed uniform dopant incorporation within the ceria lattice. The small discrepancies in oxygen content are consistent with the presence of oxygen vacancies formed to compensate for charge introduced by trivalent cations substituting for Ce^4+^ [43]. Compounds with (nearly) equimolar ratios (0.2–0.3) showed nearly ideal cation ratios and slightly oxygen-deficient composition, suggesting well-balanced defect chemistry and efficient charge neutrality. At higher dopant content (0.4–0.5), the oxygen stoichiometry deviated further, possibly due to enhanced vacancy formation and potential defect clustering [26]. Although trivalent dopant substitution in ceria generates oxygen vacancies, the experimental EDS data reveal a slight increase in oxygen stoichiometry at higher Pr fractions. This deviation arises from a combination of physical and analytical effects. At higher Pr concentrations, oxygen vacancies tend to associate with dopant cations, forming defect clusters that effectively saturate vacancy sites and suppress further vacancy generation [26,44]. Simultaneously, lattice distortion at higher Pr content enhances Ce^3+^ oxidation back to Ce^4+^ and promotes oxygen re-absorption from the ambient atmosphere, which produces an apparent oxygen enrichment [24,45]. Even though EDS analysis tends to overestimate oxygen due to matrix effect and incomplete corrections for X-ray absorption [46], which means the observed oxygen stoichiometries >2.00 do not indicate true over-stoichiometry but rather deflect clustering and partial reoxidation phenomena, which is consistent with defect saturation behavior reported in similar systems. EDS sum spectra are shown in Figure S2.

#### 2.2.2. Raman Spectroscopy

**Figure 4:**
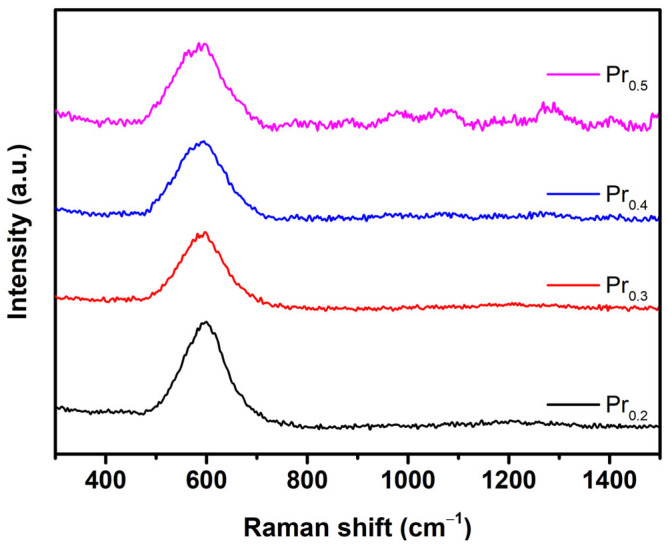
Raman spectra of as-synthesized compounds.

When discussing structural evolution, Raman spectroscopy is a powerful tool for comparative analysis. Typically, cubic CeO_2_ possesses one active, F_2g_, Raman mode, which significantly decreases with the introduction of defects in crystal lattice, while simultaneously, a new defect band appears at approx. 600 cm^−1^ [47]. In ceria-based high-entropy oxides, specifically in nanocrystalline form with increased defects, F_2g_ almost disappears, which is also visible in Figure 4 for Pr_0.2_ compound, where slight maximum is visible in the range of 420–460 cm^−1^, but this feature lowers at higher Pr concentrations. The disappearance of strong attenuation of characteristic F_2g_ band of the fluorite structure can be attributed to extensive defect formation and local symmetry breaking induced by Pr substitution. In Pr-doped ceria, the introduction of Pr^3+^/Pr^4+^ mixed valence cations lead to a higher concentration of oxygen vacancies. As the concentration of defects increases, a broad, defect-related band develops around 550–600 cm^−1^, often overlapping and effectively masking the F_2g_ feature [39]. In addition, resonance enhancement of defect-related modes involving Pr^4+^ near 530 nm excitation significantly amplify the defect band relative to F_2g_, leading to its apparent disappearance [48]. Total apparent absence of F_2g_ band at Pr_0.4_ and Pr_0.5_ can be explained by transformation of F_2g_ band into a broad background feature, which is characteristic of disordered or reduced Ce-Pr-Zr-O lattices [49]. This absence typically reflects the dominance of defect related vibrations and stronger local disorder, rather than the loss of the fluorite framework. The emergence of broad and weak Raman band between 1000 and 1600 cm^−1^ in the Pr_0.4_ and Pr_0.5_ compounds can be attributed to second order and vibronic processes associated with the strong defect–electron–phonon coupling in highly disordered lattices [50]. In defective ceria-based oxides, the principal defect-induced modes give rise to higher-frequency Raman features in the range 1000–1300 cm^−1^ [51]. Loridant [50] demonstrated that in redox active doped ceria, particularly systems with Tb or Pr, these higher frequency bands are intensified by the strong vibronic coupling between localized 4*f* localized electronic states, and lattice phonons, resulting from 4*f*–2*p* charge–transfer transitions. The appearance of these bands signifies the dominance of localized electronic transitions and small polaron like excitations, rather than simple lattice vibrations, consistent with previous reports [49]. Consequently, this region above 1000 cm^−1^ in Pr_0.4_ and Pr_0.5_ represents a spectroscopic fingerprint of extensive electronic-lattice coupling and short range structural disorder arising from the increased Pr content and oxygen vacancy concentration.

#### 2.2.3. UV–VIS Spectroscopy

Diffuse reflectance UV–Vis spectroscopy of synthesized compounds reveals direct bandgap energies in the narrow range of 2.63–2.66 eV, as determined from Tauc plot extrapolation (Figure S3). This bandgap is notably smaller than that of pure CeO_2_, additionally confirming the possibility to alter bandgap value by different dopants. As shown by Poggio-Fraccari et al. [29], Pr substitution lowers the oxygen vacancy formation energy and enhances the reducibility of Ce-Pr oxides, directly modifying their electronic density of states. Similarly, Xu et al. [38] demonstrated that Pr^3+^ ions substitute Ce^4+^ in the fluorite lattice, producing charge-compensating vacancies and intermediate 4f defect levels that extend absorption into the visible region. Furthermore, the coexistence of mixed oxidation states, such as Pr^3+/4+^ and Ce^3+/4+^, give rise to multiple optical transitions, including O 2p to Ce 4f, O 2p to Pr 4f and Pr^3+^ to Pr^4+^ charge transfers excitations [38,52,53]. These overlapping electronic transitions usually create sub-bandgap absorption tails and can appear experimentally as an apparently single, but narrowed band gap. Similar effects have been observed in high-entropy fluorite oxides containing Pr, where strong 4*f*–2*p* hybridization and oxygen-vacancy disorder progressively reduced the optical gap from 3.15 eV to 1.87 eV [53].

#### 2.2.4. X-Ray Photoelectron Spectroscopy

**Figure 5:**
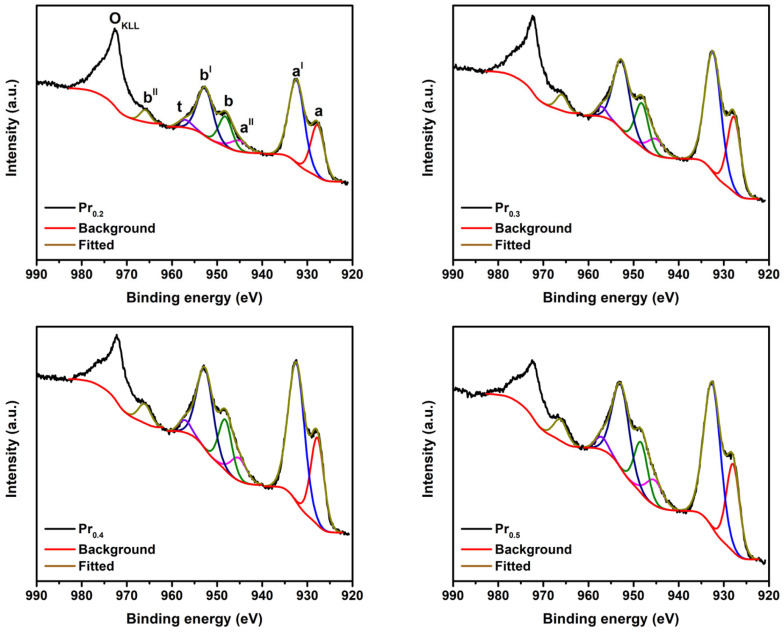
Deconvoluted Pr 3d XPS spectra of synthesized compounds.

To investigate the surface chemical composition and oxidation states of the elements present in the Pr-substituted high-entropy fluorite oxides, XPS measurements were performed. The survey spectra confirm the presence of Ce, Zr, Pr, Sm, Eu, and O, indicating the successful incorporation of all cations into the surface region (Figure S4). The C 1s peak at 284.8 eV, assigned to adventitious carbon, was used as an internal standard for binding-energy calibration (Figure S5). The Ce 3d spectra (Figure S6) display the characteristic multiplet structure composed of ten peaks associated with Ce^4+^ (u, u^II^, u^III^, v, v^II^, v^III^) and Ce^3+^ (u^I^, v^I^, u^0^, v^0^) species, consistent with the CeO_2_ fluorite structure [54,55]. Deconvolution reveals a predominant Ce^4+^ component accompanied by a minor Ce^3+^ contribution (Table 1), confirming the partial reduction in Ce^4+^ to Ce^3+^. The relative Ce^3+^ concentration increases slightly from 3.1% for Pr_0.2_ to 5.8% for Pr_0.4_, followed by a decrease to 3.2% for Pr_0.5_, suggesting that Pr incorporation promotes Ce^4+^ to Ce^3+^ reduction up to an optimal concentration. Such redox coupling between Ce and Pr is already reported in Ce-Pr mixed oxides, where Pr^3+/4+^ redox transitions facilitate oxygen-vacancy formation and alter the local electronic environment [55]. The Pr 3d region in Figure 5 exhibits overlapping doublets characteristic of mixed-valence Pr^3+^/Pr^4+^ species. The features centered around 933.1 eV and 936.2 eV correspond to Pr^3+^ 3d_5/2_ and Pr^4+^ 3d_5/2_ states, respectively, along with associated shake-up satellites [55]. Quantitative fitting indicates a progressive decrease in the relative surface Pr^3+^ fraction from 47.9% (Pr_0.2_) to 36.1% (Pr_0.5_), consistent with increasing oxidation and partial stabilization of Pr^4+^ species at higher Pr contents [55].

The O 1s spectra were deconvoluted into the following two main components: the lattice oxygen peak (O_L_) at approx. 529.4 eV and a higher-binding energy component (O_ads_) at 531.0–532.0 eV, attributed to surface hydroxyl groups, oxygen vacancies, or adsorbed oxygen species, as seen in Figure S7 [56]. The proportion of O_ads_ increases notably with Pr content, reaching 45.7% for Pr_0.5_, confirming that Pr incorporation promotes surface defect formation and enhances oxygen mobility. The Zr 3d spectrum in Figure S8 shows a single doublet (Zr^4+^ 3d_5/2_ at approx. 182.3 eV and Zr^4+^ 3d_3/2_ at approx. 184.7 eV) [2], confirming that Zr remains fully oxidized and chemically stable across all compositions. The Eu 3d and Sm 4d spectra were recorded to verify the presence of these elements at the surface and are presented in Figure S9. However, due to extensive multiplet splitting and overlapping satellite structures, reliable deconvolution proved challenging [57]. Therefore, these spectra provide qualitative confirmation of Eu and Sm incorporation at the surface, consistent with their expected trivalent states. Valence-band analysis from Figure S10 reveals that the top of the valence band lies between 0.7 and 1.5 eV below the Fermi level, indicative of a narrow band gap and defect-assisted electronic structure, consistent with the UV–Vis DRS results. The valence edge broadening with increasing Pr content reflects enhanced 4*f*–2*p* hybridization and oxygen-vacancy-related states.

### 2.3. Methylene Blue Degradation Analysis

The photocatalytic degradation performance of the synthesized high-entropy oxides was evaluated under UV illumination (*λ* = 376 nm) using methylene blue (MB) as a model organic pollutant. Although the synthesized materials possess narrow optical bandgaps (2.63–2.66 eV) that typically favor visible-light photocatalysis, they were deliberately tested under ultraviolet light to examine their intrinsic charge-transfer capability and photochemical stability under higher photon energy conditions, while pushing its limits as UV light photocatalysts, even though they originally have properties to be successful visible-light-driven photocatalysts. Visible-light-driven photocatalysis is additionally performed to confirm their photocatalytic efficiency under visible light, and without co-catalyst addition, as shown in Figure S11.

**Figure 6:**
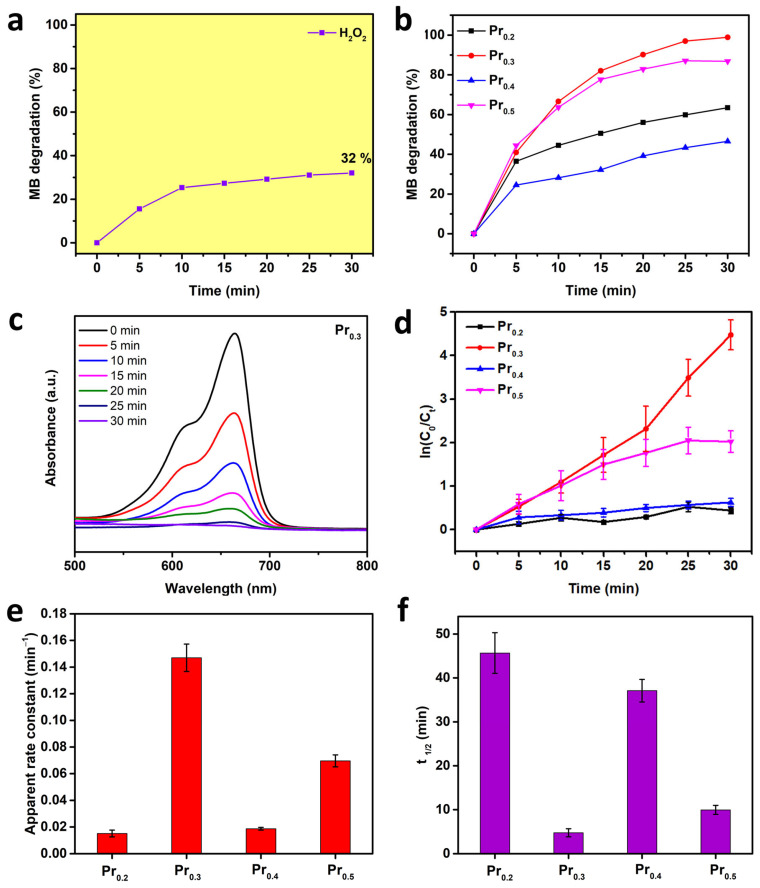
MB degradation using a pure H_2_O_2_ (**a**), and with added compounds as photocatalysts (**b**); obtained UV–Vis spectra for MB degradation using Pr_0.3_ compound over 30 min time period (**c**); ln(C_0_/C_t_) plot vs. time (**d**); apparent rate constant for tested compounds (**e**); and t_1/2_ for each tested compound (**f**).

Prior to UV-light-driven photocatalysis testing, a control experiment containing only MB and H_2_O_2_ was performed to quantify the contribution of homogeneous photolysis and direct peroxide activation (Figure 6a). Only about 32% of MB degradation was achieved after 30 min, demonstrating that light irradiation and peroxide alone are insufficient for efficient dye removal. The photocatalytic performance of all Pr-substituted samples is presented in Figure 6b. The degradation efficiency increased sharply within the first 10 min of irradiation for all catalysts, followed by a slower approach toward saturation, indicating a typical pseudo-first-order kinetic behavior [58]. Among the tested compositions, Pr_0.3_ exhibited the highest degradation efficiency (98.9%), followed by Pr_0.5_ (86.8%), Pr_0.2_ (63.4%), and Pr_0.4_ (46.5%) after 30 min of illumination. The total duration time was selected as per the first compound to reach nearly 100% degradation. This performance trend suggests that an optimal Pr concentration provides the most effective balance between oxygen vacancy density and the coexistence of Pr^3+^/Pr^4+^ and Ce^3+^/Ce^4+^ redox pairs, thereby maximizing the rate of electron-hole separation and interfacial ROS generation [59,60]. The progressive spectral evolution of MB degradation over the most active Pr_0.3_ catalyst is shown in Figure 6c. The characteristic absorption peak at 664 nm gradually decreased in intensity with increasing irradiation time, accompanied by a visible color fading of the solution from deep blue to nearly colorless. The absence of significant spectral shifts indicates that the degradation process primarily involves stepwise demethylation and ring-opening oxidation, typical of MB mineralization under oxidative conditions [61].

To gain deeper insight into reaction dynamics and quantify relative catalytic activities, the kinetics of MB degradation were analyzed using the pseudo-first-order Langmuir–Hinshelwood model [58]. The linear relationships between ln(C_0_/C_t_) and irradiation time for all samples are presented in Figure 6d. All curves exhibited excellent linearity, confirming that the degradation process follows pseudo-first-order kinetics, with the reaction rate directly proportional to the surface concentration of adsorbed MB molecules [2]. The apparent rate constants (*k*_app_), obtained from the slope of the linear fits, are compared in Figure 6e. The rate constants correspond to the observed degradation efficiencies [59]. The Pr_0.3_ compound reached the highest apparent rate constant (0.15 min^−1^), nearly an order of magnitude higher than that of Pr_0.2_, indicating that the optimum Pr substitution promotes rapid electron-hole separation and more efficient formation of reactive oxygen species. The calculated half-life times (t_1/2_ = ln(2)/*k*_app_) for each composition are displayed in Figure 6f. The shortest t_1/2_ was recorded for Pr_0.3_, whereas Pr_0.2_ and Pr_0.4_ required substantially longer times, in agreement with their lower rate constants.

**Figure 7:**
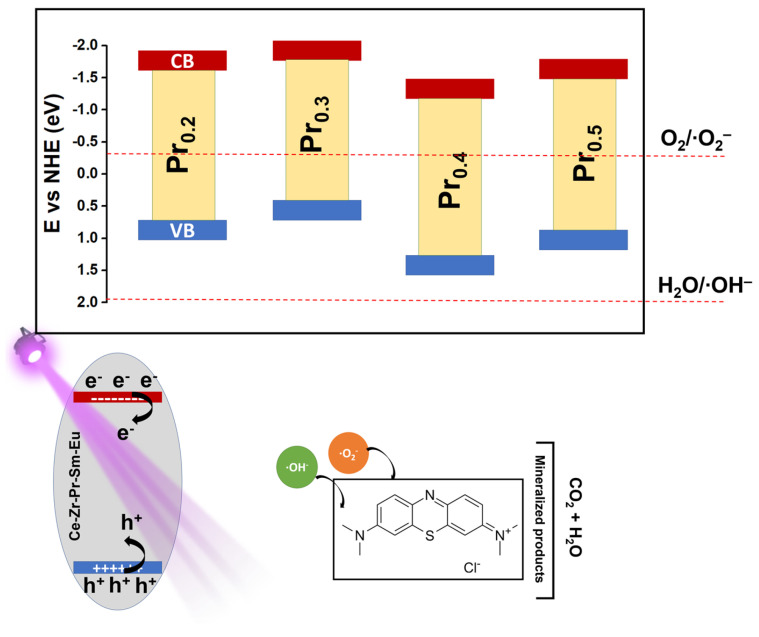
Proposed photocatalytic mechanism of MB degradation.

Based on the optical, structural, and photocatalytic results, as shown in Table 2, a mechanism for MB degradation over the synthesized high-entropy fluorite oxides has been proposed (Figure 7). The measured optical bandgaps and valence band maxima indicate that these oxides possess narrowed electronic structures, as compared to pure CeO_2_ [2], primarily due to the incorporation ions with variable oxidation states.

This mixed valence introduces intermediate 4*f* defect levels and oxygen-vacancy-related subbands, thereby promoting visible-light absorption and facilitating charge transfer across the lattice [38]. Upon UV illumination, electrons in the valence band (VB) are excited to the conduction band (CB), leaving behind photogenerated holes [62]. The CB edge positions (−1.72, −1.96, −1.15, and −1.58 eV for Pr_0.2_, Pr_0.3_, Pr_0.4_, and Pr_0.5_, respectively) are sufficiently negative to thermodynamically enable the reduction in dissolved or adsorbed molecular oxygen to superoxide radicals (∙O_2_^−^). In contrast, the VB maxima (0.69–1.51 eV vs. NHE) are not positive enough to directly oxidize water or hydrogen peroxide to hydroxyl radicals (∙OH) [63,64]. Therefore, direct ∙OH formation from water oxidation is unlikely in these systems. Instead, hydroxyl radicals can predominantly be generated via photo-assisted Fenton-like activation of H_2_O_2_. The photogenerated electrons are rapidly trapped by Ce^4+^ or Pr^4+^ species, forming Ce^3+^ and Pr^3+^ ions, while suppressing electron-hole recombination. These reduced metal centers react with H_2_O_2_ to generate hydroxyl radicals and regenerate the higher oxidation states, establishing a catalytic redox cycle. Simultaneously, electrons from CB can participate in reductive activation of H_2_O_2_, further contributing to ROS formation. This dual redox cycling, sustained by the coexistence of 4+/3+ pairs, enhances carrier separation and continuously regenerates active species on the catalyst surface [65,66,67].

The redox coupling between adjacent Pr and Ce centers, acts as an internal electron relay, minimizing charge recombination and supporting a quasi-Fenton cycle that promotes continuous ROS production [48,68]. The superoxide radicals produced via oxygen reduction can further evolve through secondary reactions [69]. The combined action of superoxide and H_2_O_2_ derived hydroxyl radicals constitutes the dominant oxidative pathway responsible for the degradation. The generated radicals attack the conjugated chromophore of MB, causing progressive demethylation, ring-opening, and eventual mineralization to CO_2_, NH_4_^+^, and SO_4_^2−^ [69,70,71,72]. In this mechanism, the oxygen vacancies introduced by Pr substitution serve as active adsorption sites for O_2_ and H_2_O_2_ molecules, lower the activation barrier for radical formation, and promote fast electron transfer through localized defect bands. The structural flexibility of the high-entropy fluorite lattice allows for reversible redox transitions without loss of crystallinity or catalytic performance, ensuring sustained activity during repeated photocatalytic cycles. In general, mechanism can be summarized as a multi-step photo-Fenton-like process driven by mixed-valence rare-earth redox couples in the next order: (i) generation of e^−^/h^+^ pairs in the HEO under UV irradiation; (ii) electron transfer through Ce^4+/3+^ and Pr^3+/4+^ cycles enhances separation; (iii) surface reactions with O_2_ and H_2_O_2_ produce ∙O_2_^−^ and ∙OH radicals; (iv) radicals attack MB molecules, leading to chromophore cleavage and mineralization; and (v) redox couples are continuously restored through electron exchange with oxygen and peroxide species.

## 3. Materials and Methods

### 3.1. Synthesis and Materials

**Figure 8:**
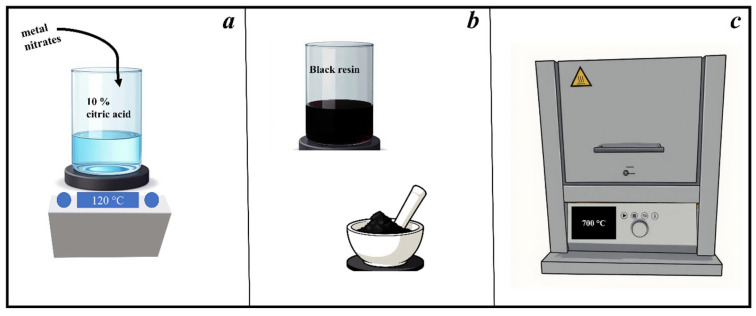
Schematic presentation of the synthesis procedure showing (**a**) addition of metal nitrates to previously prepared 10% citric acid solutions, and then heated and mixed at 120 °C, (**b**) formed black resin that was grounded in mortar, and then followed by (**c**) calcination at 700 °C.

High-entropy oxides were synthesized via a modified aqueous sol–gel citrate route, adapted from previously established procedures for complex oxide systems, and shown in Figure 8 [73]. Stoichiometric amounts of metal nitrates corresponding to the targeted compositions presented in Table 3 (Ce, Zr, Sm, Eu, and Pr ratios with the total cationic sum fixed at 1 mmol) were used to achieve high configurational entropy in the system. In a typical synthesis, a total of 1 mmol of mixed metal precursors was dissolved in a 10% aqueous citric acid solution (prepared by dissolving 10 g of citric acid in 100 mL of Milli-Q water) under constant magnetic stirring. The resulting solution was stirred for 30 min to ensure complete dissolution of all precursors, after which the pH value was adjusted to approximately 5 by the slow addition of concentrated ammonia solution, monitored with a pH meter (HANNA Instruments, Zagreb, Croatia). The reaction mixture was then stirred and heated at 120 °C until solvent evaporation produced a black resin. This resin was dried overnight at 120 °C in an oven (Instrumentaria ST-01/02, Sesvete, Croatia) to remove residual moisture. The dried mass was ground manually using a mortar and pestle and then subjected to calcination in a muffle furnace (Nabertherm GmbH, Lilienthal, Germany) at 700 °C for 8 h, with a controlled heating rate of 240 °C per hour. The calcination process yielded single-phase, high-entropy oxide powders with homogeneous distribution of constituent cations. Chemical used for the synthesis were used as purchased: Ce(NO_3_)_3_ ∙ 6H_2_O, Eu(NO_3_)_3_ ∙ 5H_2_O, Pr(NO_3_)_3_ ∙ 6H_2_O, ZrO(NO_3_)_2_ ∙ *n*H_2_O, purchased from Sigma Aldrich, Taufkirchen, Germany; Sm(NO_3_)_3_ ∙ 6H_2_O purchased from ThermoFisher, Waltham, MA, USA; Citric acid monohydrate purchased from TTT, Sveta Nedelja, Croatia; and concentrated ammonia solution from Gram-Mol, Zagreb, Croatia.

### 3.2. Structural Characterization

Powder X-ray diffraction (PXRD) analysis was employed to examine the crystal structure and microstructure of the synthesized materials. Measurements were conducted using a PANalytical Aeris Research Diffractometer (PANalytical, Malvern, UK) operating in *θ-θ* geometry with Cu *K*α radiation (*λ* = 1.5406 Å) generated at 40 kV and 15 mA. Data were collected over a 2*θ* range of 20–100° at 25 °C, with a step size of 0.02° and a counting time of 20.4 s/step. Instrumental parameters included a divergence slit of 1° and a fixed mask of 13 mm. The obtained diffraction patterns were refined using the Rietveld method implemented in the FULLPROF software suite (version: January-2021) [74], applying the Thomson–Cox–Hastings pseudo-Voigt profile function to ensure accurate phase quantification and structural modeling. Visualization of the refined structures were performed using the VESTA software package (version: 3.4.8) [75].

The surface microstructure of the samples was characterized using a Thermo Fisher Scientific Apreo C field-emission scanning electron microscope (Thermo Fisher Scientific, Waltham, MA, USA).

### 3.3. Spectroscopy Analysis

Elemental distribution analysis was carried out using an Oxford Instruments X-Max^50^ microanalysis system (Oxford Instruments plc, Tubney Woods, Abingdon, UK) equipped with a 50 mm^2^ silicon drift detector (SDD). The corresponding elemental maps were acquired and processed using INCA software (INCA V7.5, Oxford Instruments plc, Tubney Woods, Abingdon, UK), enabling visualization of the spatial distribution and homogeneity of the constituent elements within the samples.

Raman spectra were acquired using a Bruker Senterra II Raman spectrometer (Bruker, Billerica, MA, USA) equipped with a 532 nm excitation laser. All samples were measured under identical conditions with a laser power of 2.5 mW, an integration time of 10,000 ms and averaged over three co-additions to improve the signal-to-noise ratio. The spectrometer has a spectral resolution of 4 cm^−1^.

X-ray photoelectron spectroscopy (XPS) measurements were performed on a Kratos Supra+ spectrometer (Kratos Analytical, Manchester, UK) equipped with a monochromatic Al Kα radiation source (*hν* = 1486.6 eV). To prevent surface charging during analysis, a low-energy charge neutralizer was employed throughout all measurements. Spectra were collected at a take-off angle of 90° relative to the sample surface, using a pass energy of 20 eV for high-resolution scans. Powder samples were pressed onto conductive carbon adhesive tape and mounted on a silicon wafer for analysis. Data acquisition and processing were performed using ESCApe 1.5 software (Kratos Analytical). All binding energies were calibrated with respect to the C 1s hydrocarbon peak (C–C/C–H) set at 284.8 eV to correct for any residual surface charging effects.

The specific surface area and pore characteristics of the synthesized oxides were determined using a gas sorption analyzer Autosorb iQ (Quantachrome Instruments, Boynton Beach, FL, USA). Each sample was degassed at 250 °C under vacuum for 5 h using a degassing station to remove adsorbed moisture and residual impurities from the pores. Nitrogen adsorption–desorption isotherms were recorded at −196 °C. The obtained isotherms were used to calculate the specific surface area of the mesoporous materials according to the Brunauer–Emmett–Teller (BET) method.

### 3.4. Methylene Blue Degradation Analysis

Experiments on the photocatalytic degradation of organic dyes were carried out at room temperature under a UV lamp, which exposed the samples to light with a wavelength of 376 nm. The lamp was kept at a constant distance from the samples. A stock solution of methylene blue (MB) with a concentration of 2 × 10^−5^ mol/L was prepared in ultrapure water. All reactions were conducted in a 50 mL laboratory beaker, and the suspensions were stirred during light exposure using a magnetic stirrer. To 20 mL of the stock MB solution, 2 mg of the synthesized compounds and 4 drops of the cocatalyst H_2_O_2_ were added, and the resulting suspensions were subjected to constant stirring and illumination. Control experiments were performed by adding only H_2_O_2_ to the MB solution in order to analyze MB degradation exclusively under illumination and in the presence of the cocatalyst. Afterwards, the degradation of MB was monitored using a UV–Vis spectrophotometer (UV-3600Plus, Shimadzu, Kyoto, Japan) by taking aliquots of the reaction mixture at regular 5 min intervals. Absorbance was measured at 664 nm, which corresponds to the maximum absorption wavelength of MB, and a total reaction time was used to be the time the first compound reached a nearly 100% degradation. Each photocatalytic degradation experiment was performed in triplicate under identical conditions, and the reported values represent the average of three independent measurements, while the corresponding standard deviations are shown as error bars where applicable.

Additionally, visible light-driven photodegradation of methylene blue was conducted under the same conditions as described above, but without co-catalyst addition, and under 20 W tungsten-halogen lamp to confirm the photocatalytic efficiency under visible light.

**Figure 9:**
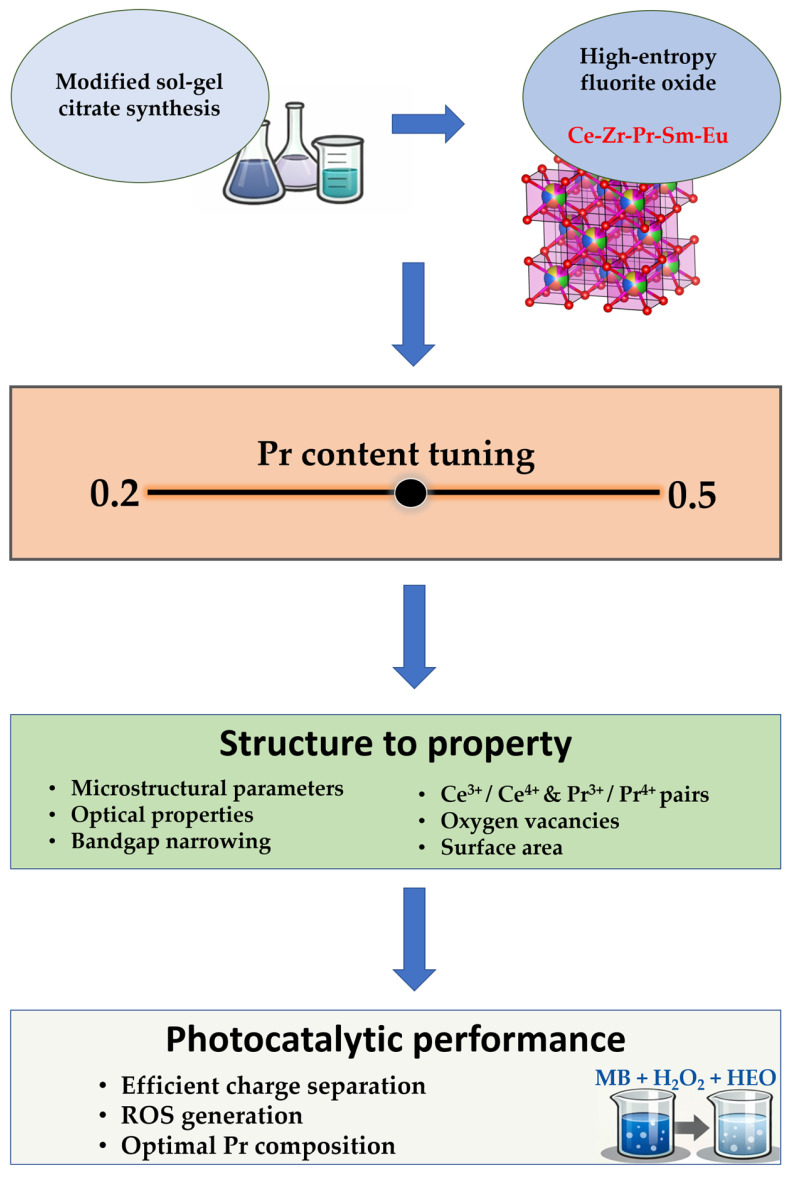
The flowchart of experimental operation process.

The overall experimental logic of this research is summarized in Figure 9.

## 4. Conclusions

This study systematically elucidates the influence of praseodymium incorporation on the structural evolution, defect chemistry, and photocatalytic activity of Ce-Zr-Pr-Sm-Eu-based high-entropy fluorite oxides. Through careful compositional control and comprehensive structural characterization, it was demonstrated that Pr substitution profoundly alters the balance between lattice disorder, oxygen vacancy formation, and redox flexibility. All synthesized compositions crystallized in a single-phase cubic fluorite structure, confirming that high configurational entropy successfully stabilizes complex multicomponent solid solutions without phase segregation. Progressive lattice expansion and increased microstrain with rising Pr content were directly correlated with the incorporation of larger Pr^3+^ ions and the generation of compensating oxygen vacancies. Morphological and physisorption analyses revealed that moderate Pr concentrations favored the formation of highly porous nanostructures with abundant surface-active sites, whereas excessive doping led to partial densification due to defect clustering and enhanced cation diffusion during calcination. Spectroscopic characterization provided deeper insight into the defect-redox behavior. Raman and XPS analyses confirmed the coexistence of Pr^3+^/Pr^4+^ and Ce^3+^/Ce^4+^ species, illustrating strong redox coupling between the two cationic sublattices. This coupling enhances electronic communication through hybridization, creating intermediate defect-related electronic states that facilitate charge delocalization and bandgap narrowing. The presence of abundant surface oxygen vacancies, confirmed by O 1s XPS deconvolution, enhances improved oxygen mobility and redox reversibility. The UV–Vis spectra corroborated these findings, showing reduced optical bandgaps indicative of defect-mediated electronic transitions and strong hybridization effects. Photocatalytic degradation experiments using methylene blue as a model organic pollutant revealed that Pr concentration determines the efficiency of light-driven reactions. The optimal composition, with moderate Pr substitution, exhibited the highest degradation efficiency, demonstrating that a balanced defect population maximizes charge-carrier separation and reactive oxygen species generation. The photocatalytic process proceeds via a photo-Fenton-like mechanism driven by redox cycling between Ce^4+/3+^ and Pr^3+/4+^ redox couples, leading to efficient dye degradation and mineralization. In general, prior highlight of this research is the fact that typically UV inactive photocatalysts can achieve higher photocatalytic efficiency by altering their properties and/or constituent ratio, remaining stable in its high-entropy form.

## Figures and Tables

**Table 1:** Obtained surface at. concentration of Ce^3+^, Pr^3+^, and adsorbed oxygen species from deconvolution of XPS spectra.

Compound	Ce^3+^/Ce^4+^ (%)	Surface Atomic Concentration (%) [Ce^3+^]	Surface Atomic Concentration (%) [Pr^3+^]	Surface Atomic Concentration (%) [O_ads_]
Pr_0.2_	3.1	2.9	47.9	33.9
Pr_0.3_	5.6	5.3	44.1	28.2
Pr_0.4_	5.8	5.5	41.7	43.1
Pr_0.5_	3.2	3.1	36.1	45.7

**Table 2:** Overall results of tested compounds for MB degradation, along with their structural features.

Compound	MB Degradation (%)	Bandgap Value (eV)	Surface Area (m^2^/g)	Surface Atomic Concentration (%) [Pr^3+^]	Surface Atomic Concentration (%) [O_ads_]
Pr_0.2_	63.44	2.63	33.784	47.9	33.9
Pr_0.3_	98.86	2.65	29.766	44.1	28.2
Pr_0.4_	46.51	2.66	32.921	41.7	43.1
Pr_0.5_	86.77	2.66	25.925	36.1	45.7

**Table 3:** Chemical compositions of synthesized compounds.

Name of the Compound	Chemical Formula
Pr_0.2_	Ce_0.2_Zr_0.2_Pr_0.2_Sm_0.2_Eu_0.2_O_2_
Pr_0.3_	Ce_0.175_Zr_0.175_Pr_0.3_Sm_0.175_Eu_0.175_O_2_
Pr_0.4_	Ce_0.15_Zr_0.15_Pr_0.4_Sm_0.15_Eu_0.15_O_2_
Pr_0.5_	Ce_0.125_Zr_0.125_Pr_0.5_Sm_0.125_Eu_0.125_O_2_

## Data Availability

The data presented in this study are available on request from the corresponding author.

## Supplementary Materials

The following supporting information can be downloaded at https://www.mdpi.com/article/10.3390/molecules31040598/s1, Table S1: Crystallographic data obtained by Rietveld refinement; Figure S1: Physisorption isotherms of synthesized Pr_0.2–0.5_ compounds; Table S2: Data obtained by physisorption analysis; Table S3: Quantitative composition of each synthesized compound by EDS; Figure S2: EDS sum spectra of synthesized compounds; Figure S3: Tauc plots of synthesized compound for bandgap approximation; Figure S4: XPS survey spectra of as-synthesized compounds; Figure S5: Deconvoluted C 1s XPS spectra; Figure S6: Deconvoluted Ce 3d XPS spectra; Figure S7: Deconvoluted O 1s XPS spectra; Figure S8: Deconvoluted Zr 3d spectra; Figure S9: Eu 3d (left) and Sm 4d (right) XPS spectra; Figure S10: Determination of Valence band position by XPS; Figure S11: Visible light driven photocatalysis over synthesized compounds, using a 20 W halogen lamp as a light source.
