# Supplementary material for: Influence of Pr Content on Structural Evolution of Doped Ceria-Based High-Entropy Oxides
Source: Molecules. 2026 Feb 9;31(4):598. doi: 10.3390/molecules31040598 (PMC12943474; doi:10.3390/molecules31040598)
Supplement: Supplementary file 1 [file molecules-31-00598-s001.zip › molecules-4125475-supplementary.pdf]

SUPPORTING INFORMATIONS

Influence of Pr Content on Structural Evolution of Doped Ceria-Based High-Entropy Oxides

Dalibor Tatar [1], Jakov Babić [1], Stjepan Šarić [1], Jelena Kojčinović [1], Petra Šušak [1], Anamarija Stanković [1], Laura Milišić [2], Andraž Mavrič [2], Cora Deák [3], Gergő Ballai [3], Imre Szenti [3], Ákos Kukovecz [3] and Igor Djerdj [1,*]

[1] Department of Chemistry, Josip Juraj Strossmayer University of Osijek, Cara Hadrijana 8/A, HR-31000 Osijek, Croatia; dtatar@kemija.unios.hr (D.T.); jakov.babic@kemija.unios.hr (J.B.); stjepan.saric@kemija.unios.hr (S.Š.); jbijelic@kemija.unios.hr (J.K.); petra.susak@kemija.unios.hr (P.Š.); aster@kemija.unios.hr (A.S.)

[2] Materials Research Laboratory, University of Nova Gorica, Vipavska 13, SI-5000 Nova Gorica, Slovenia; laura.milisic@ung.si (L.M.); andraz.mavric@ung.si (A.M.)

[3] Department of Applied and Environmental Chemistry, University of Szeged, Rerrich Béla Sq. 1, H-6720 Szeged, Hungary; corapravda@gmail.com (C.D.); ballaigergo@gmail.com (G.B.); szentiimre@gmail.com (I.S.); kakos@chem.u-szeged.hu (Á.K.)

* Correspondence: igor.djerdj@kemija.unios.hr

Table S1. Crystallographic data obtained by Rietveld refinement.

Compound	$Pr_{0.2}$	$Pr_{0.3}$	$Pr_{0.4}$	$Pr_{0.5}$
Space Group	*Fm*-3*m*			
Crystal system	Cubic			
Data collection range	20-100°			
Phase composition (wt %)	100			
Molecular weight	161.57	156.57	163.52	169.05
Z	8	8	8	8
Lattice parameters (Å)	*a* = 5.4205 (2)	*a* = 5.4294 (2)	*a* = 5.4303 (2)	*a* = 5.4376 (2)
Cell volume ($Å^3$)	159.261 (6)	160.051 (6)	160.131 (6)	160.776 (6)
Calculated density (g/cm^3)	7.126	7.301	7.141	6.986
No. of parameters refined	16	16	16	16
Average crystallite size (nm)	10	10	12	12
Average microstrain (%)	0.3315	0.3520	0.2827	0.3290
R_p; R_{wp}; R_e (%)	21,4; 14; 12,6	18.5; 12.7; 10.6	15.2; 10; 9.02	23.9; 17.6; 10.3
R_B(%)	4.2	4.14	3.05	7.02
GoF	1.107	1.200	1.112	1.706

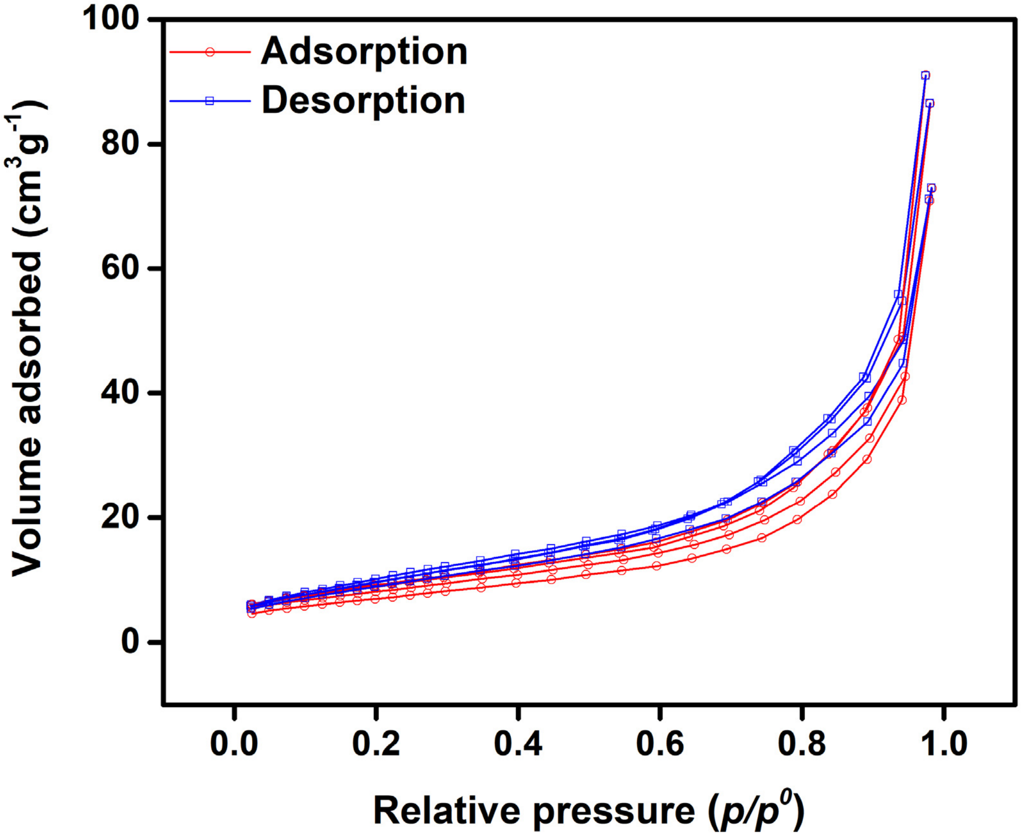

Figure S1. Physisorption isotherms of synthesized $Pr_{0.2\text{-}0.5}$ compounds.

Table S2. Data obtained by physisorption analysis.

Compound	B.E.T. (m^2g^{-1})	Pore size (nm)	Pore volume (cm^3/g)
CeO_2	21.511	12.08	0.065
$Pr_{0.2}$	33.784	5.54	0.134
$Pr_{0.3}$	29.766	7.72	0.113
$Pr_{0.4}$	32.921	9.11	0.141
$Pr_{0.5}$	25.925	3.79	0.110

Table S3. Quantitative composition of each synthesized compound by EDS.

Compound	Element	Theoretical stoichiometric ratio	Experimental stoichiometric ratio
$Pr_{0.2}$	Ce	0.2	0.23
	Zr		0.17
	Sm		0.22
	Eu		0.21
	Nd	0.2	0.22
	O	2	1.93
$Pr_{0.3}$	Ce	0.175	0.17
	Zr		0.15
	Sm		0.17
	Eu		0.16
	Nd	0.3	0.3
	O	2	2.04
$Pr_{0.4}$	Ce	0.15	0.15
	Zr		0.12
	Sm		0.14
	Eu		0.13
	Nd	0.4	0.39
	O	2	2.06
$Pr_{0.5}$	Ce	0.125	0.12
	Zr		0.1
	Sm		0.12
	Eu		0.11
	Nd	0.5	0.49
	O	2	2.07

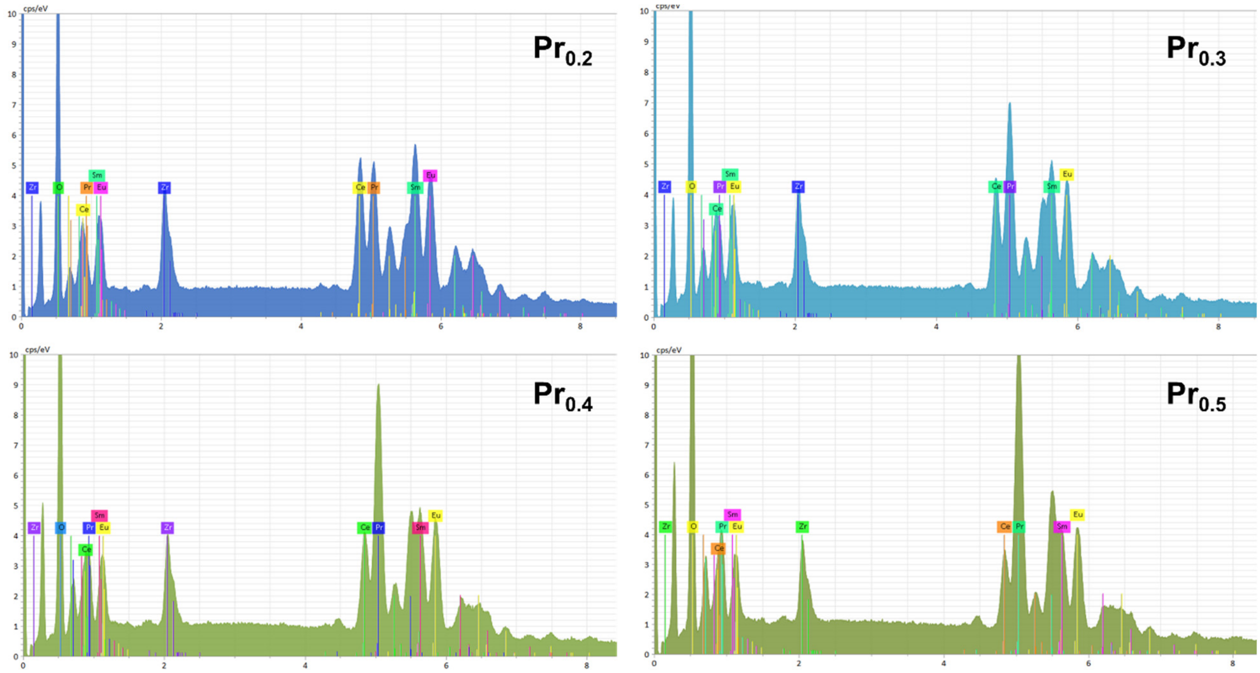

Figure S2. EDS sum spectra of synthesized compounds.

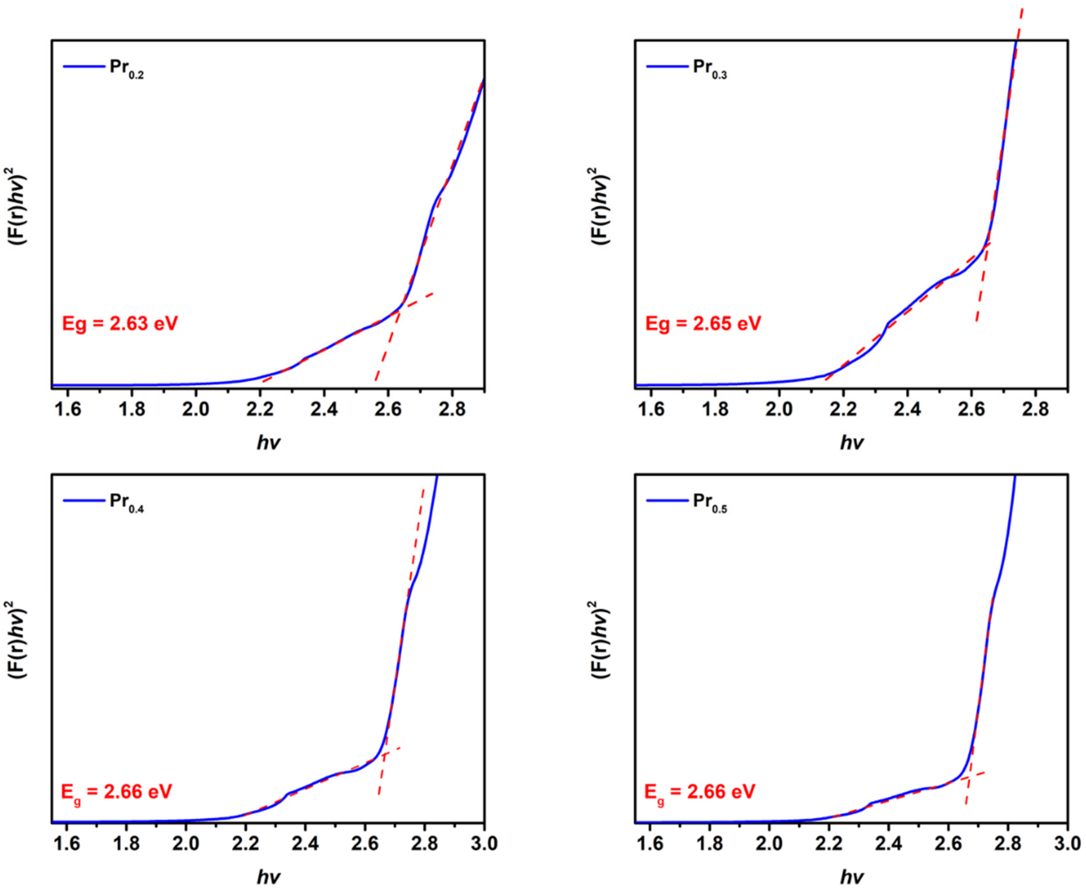

Figure S3. Tauc plots of synthesized compound for bandgap approximation.

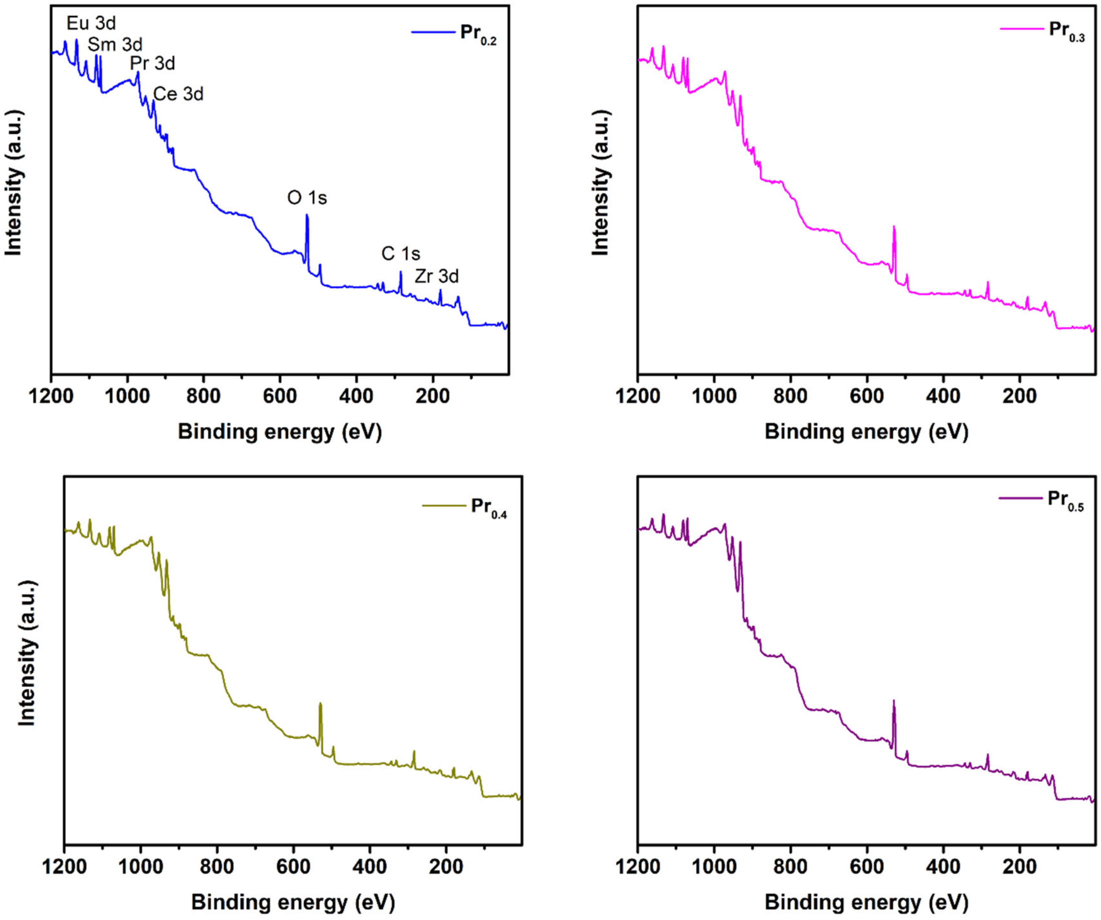

Figure S4. XPS survey spectra of as-synthesized compounds.

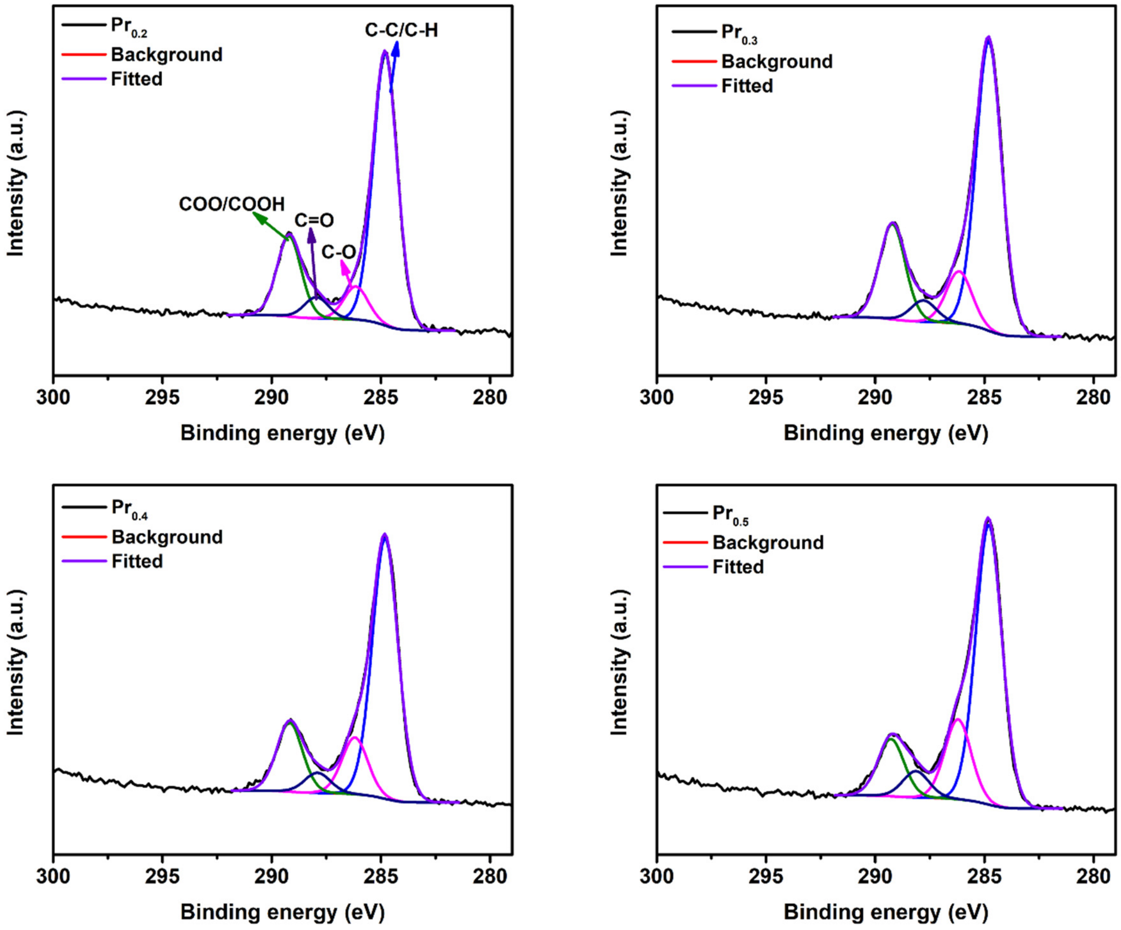

Figure S5. Deconvoluted C 1s XPS spectra.

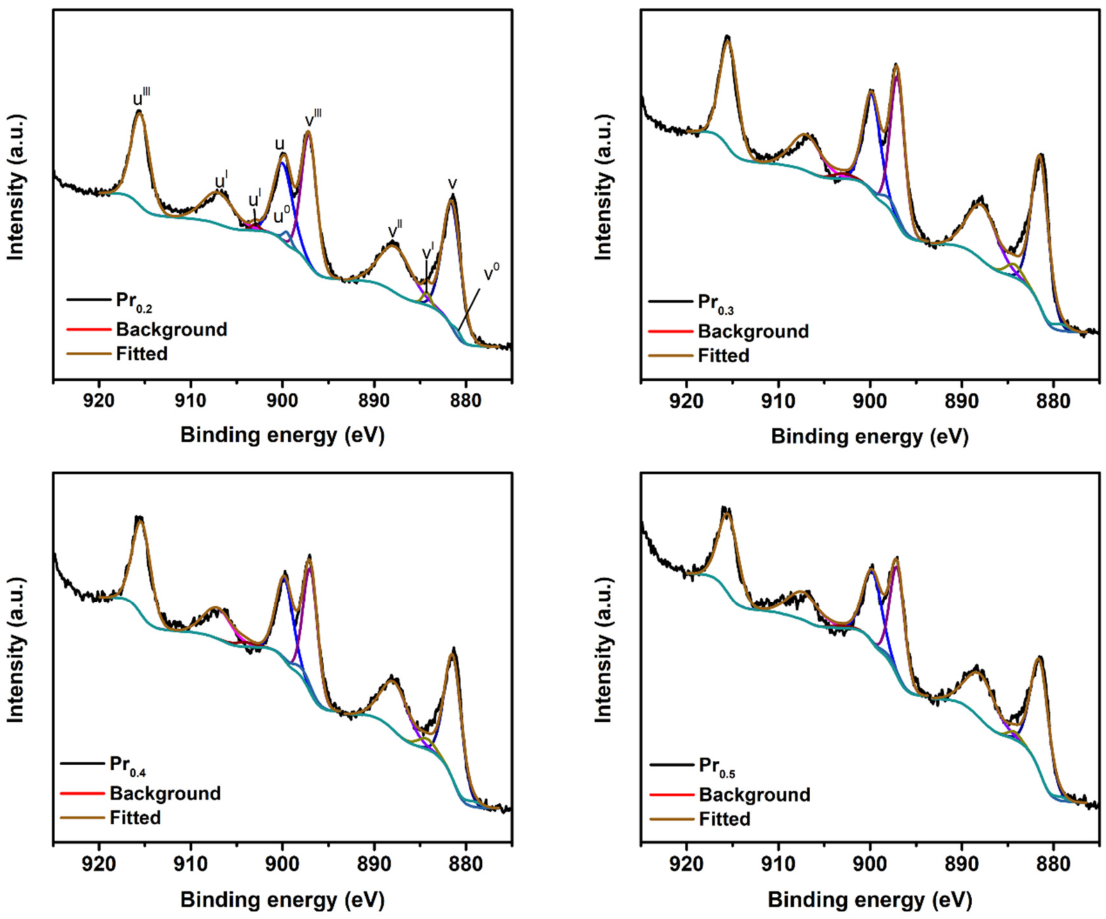

Figure S6. Deconvoluted Ce 3d XPS spectra.

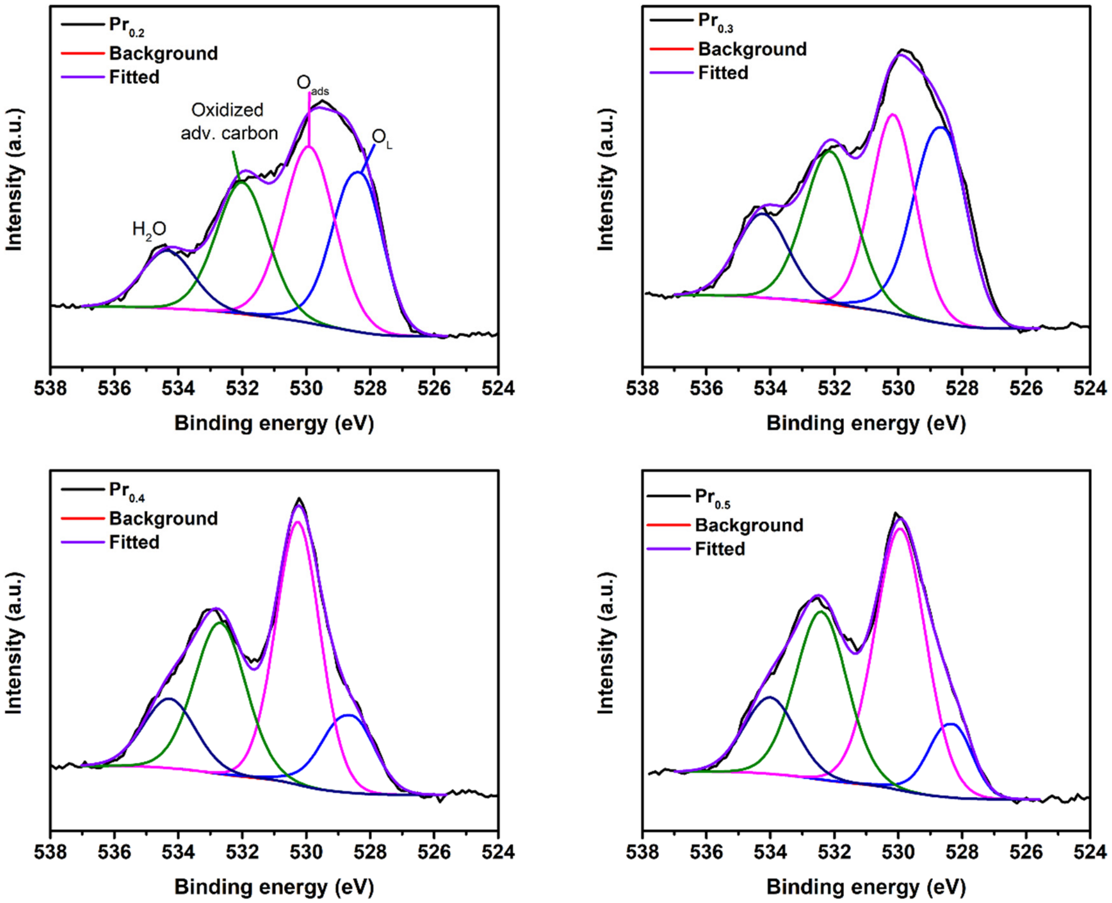

Figure S7. Deconvoluted O 1s XPS spectra.

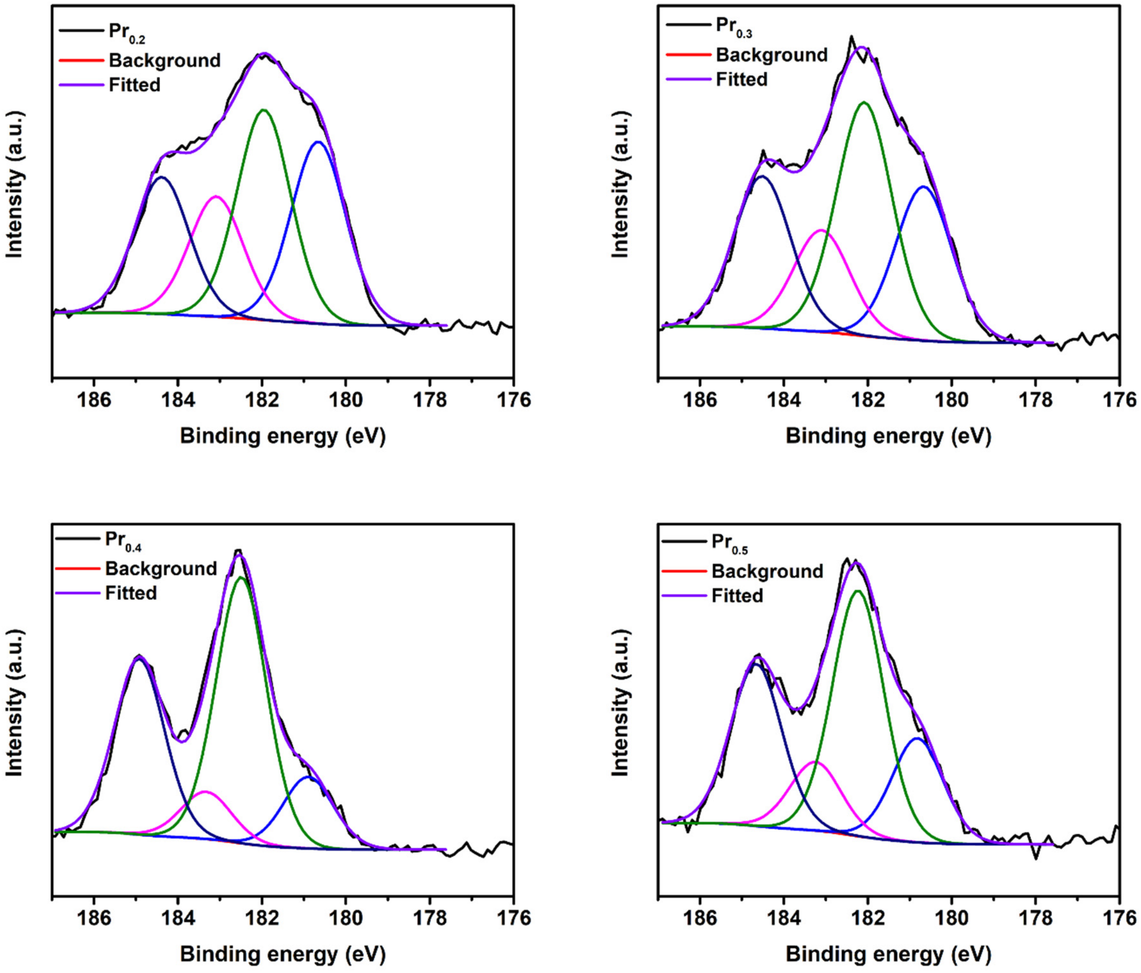

Figure S8. Deconvoluted Zr 3d spectra.

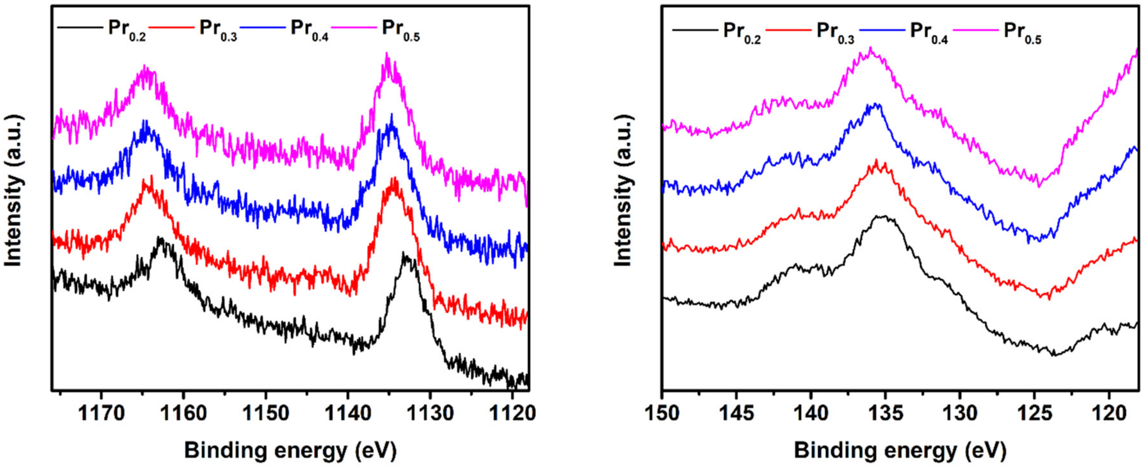

Figure S9. Eu 3d (left) and Sm 4d (right) XPS spectra.

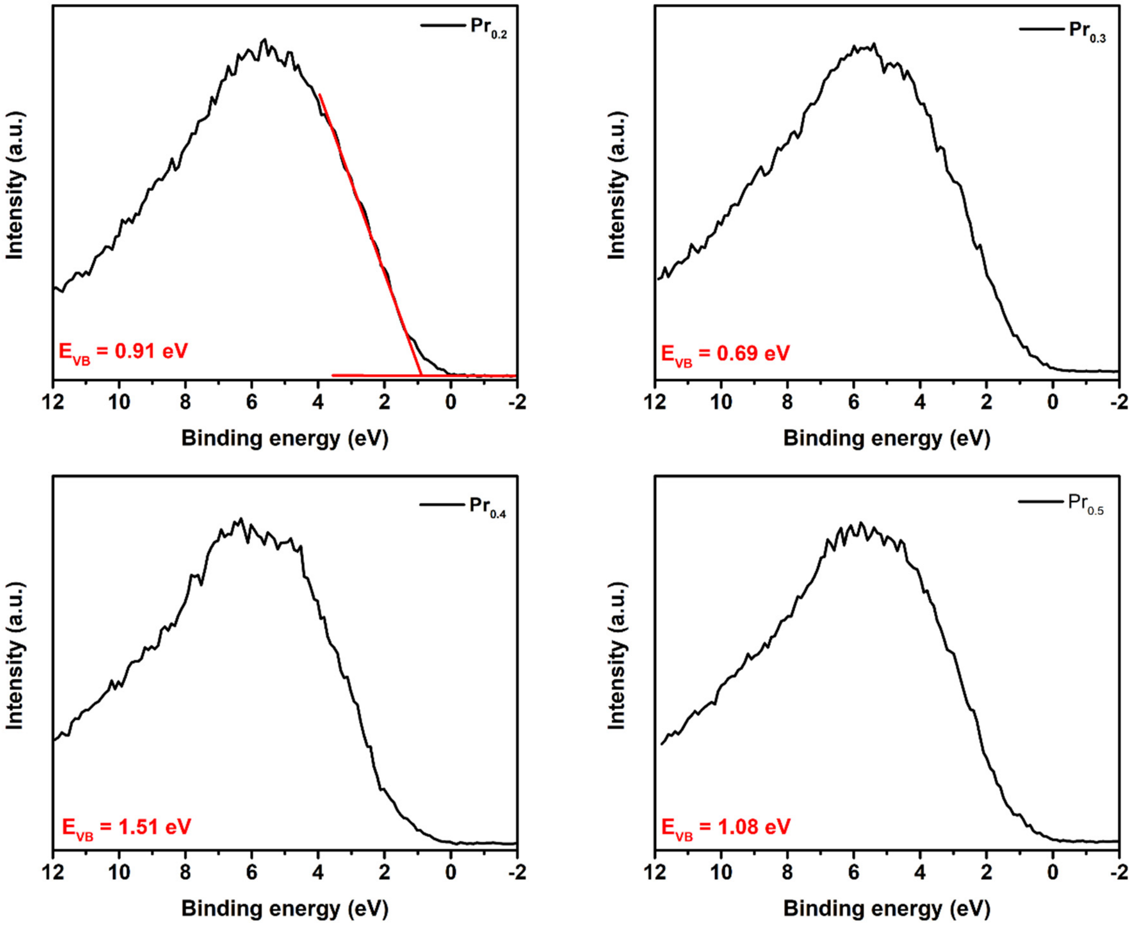

Figure S10. Determination of Valence band position by XPS.

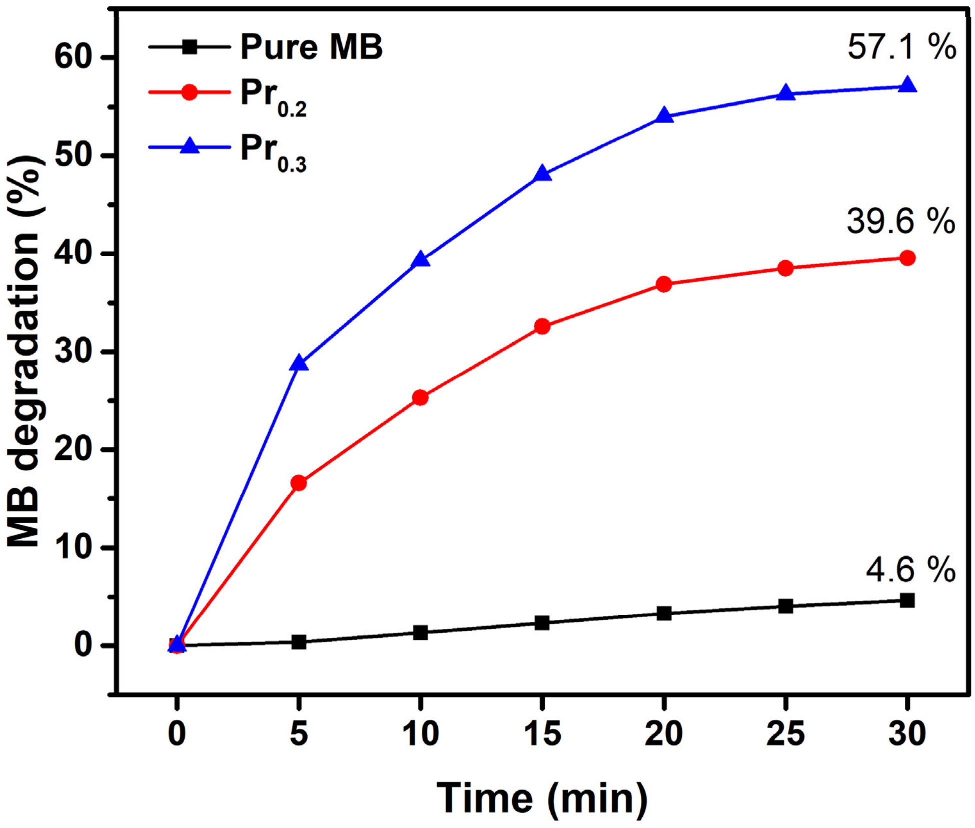

Figure S11. Visible light driven photocatalysis over synthesized compounds, using a 20 W halogen lamp as a light source.
